# Role of human papillomavirus in laryngeal squamous cell carcinoma: A meta‐analysis of cohort study

**DOI:** 10.1002/cam4.2712

**Published:** 2019-11-15

**Authors:** Huanhuan Wang, Jinlong Wei, Bin Wang, Lingbin Meng, Ying Xin, Lihua Dong, Xin Jiang

**Affiliations:** ^1^ Department of Radiation Oncology The First Hospital Jilin University Changchun China; ^2^ Department of Internal Medicine Florida Hospital Orlando FL USA; ^3^ Key Laboratory of Pathobiology Ministry of Education Jilin University Changchun China

**Keywords:** disease‐free survival (DFS), human papilloma virus (HPV), laryngeal cancer, laryngeal squamous cell carcinoma (LSCC), overall survival (OS), squamous cell cancer (SCC)

## Abstract

**Objective:**

To evaluate the association of human papilloma virus (HPV) infection with prognosis, specifically overall survival (OS) and disease‐free survival (DFS), in laryngeal squamous cell carcinoma (LSCC) patients.

**Method:**

A systematic review and meta‐analysis were performed according to the Preferred Reporting Items for Systematic Reviews and Meta‐Analyses (PRISMA) guidelines. LSCC was confirmed on the basis of histopathology, whereas HPV status was confirmed by polymerase chain reaction.

**Results:**

A total of 6539 articles were initially searched from 8 databases, of which 11 studies were eligible for our review. A total of 1442 LSCC cases were included in this analysis. Eight studies examined 3‐year OS for LSCC. The pooled hazard ratio (HR) from the 8 studies was 0.29 (95% CI: 0.25‐0.33). There was a statistically significant difference in 3‐year OS between the HPV‐negative and ‐positive groups, with the latter having a better survival. There was no statistically significant differences in 5‐ and 10‐year OS. Five studies examined 3‐ and 5‐year DFS for LSCC, whereas only 3 studies examined 10‐year DFS. There was no statistically significant difference in 3‐, 5‐, and 10‐year DFS between the HPV groups.

**Conclusion:**

This study evaluated the survival impact of HPV infection in LSCC patients. The OS of the HPV‐positive group was better than that of the HPV‐negative group in terms of short‐term survival. Compared with the HPV‐negative group, the HPV‐positive group had a better trend of DFS, suggesting that a larger sample size and further exploration of the pathology and local control of HPV‐positive tumors are needed.

## Graphical abstract

Human papillomavirus (HPV) infection is a cause of squamous cancers of the oropharynx (particularly cancers of the tonsils and tongue base), and emerging evidence showed that HPV infection may also be associated with an increased risk of laryngeal squamous cell carcinoma (LSCC). LSCC was increasingly reported to be associated with HPV infection, but the role of HPV in this type of cancer has not been conclusively established. The association of better prognosis with HPV positivity is controversial. Different test methods may result in inconsistent results, which may obfuscate the correlation between HPV status and prognosis in LSCC.

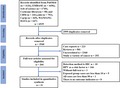

## 1 INTRODUCTION

Laryngeal squamous cell carcinoma (LSCC) represents one of the most common head and neck malignancies, accounting for approximately 20% of all cases, with up to 40% of patients presenting with advanced disease at the time of diagnosis. More than 150 000 new patients have been diagnosed worldwide and 90 000 die each year.^1^.In recent years, the incidence of LSCC has increased.

Like other head and neck cancers (HNC), LSCC has been clearly linked to smoking and alcohol consumption. The risk factors of LSCC are changing, as the prevalence of tobacco‐induced cancers has decreased and the prevalence of cancers not associated with tobacco has increased. Human papillomavirus (HPV) infection is a cause of squamous cancers of the oropharynx (particularly cancers of the tonsils and tongue base),^2^, ^3^, ^4^ and emerging evidence showed that HPV infection may also be associated with an increased risk of LSCC.^5^ The infection rate of HPV has been established to range between 3% and 85% of laryngeal cancer.^6^, ^7^ HPV types 16, 18, and 31 are the majority risk factors, and patients with HPV‐associated HNC tend to be of young age.^4^, ^8^ Currently, oropharyngeal cancers have been divided into two distinct diseases on the basis of HPV presence or absence, but there is no detailed stratification of HPV infection in LSCC. Although LSCC was increasingly reported to be associated with HPV infection,^5^, ^9^ the role of HPV in this type of cancer has not been conclusively established.^10^

All 120 HPV genotypes had been detected in squamous carcinoma, and they were classified into low‐ and high‐risk groups according their oncogenesis potential.^11^ Some clinical trials reported that patients with HPV‐positive oropharynx cancers showed better survival than those with HPV‐negative oropharynx cancers.^12^, ^13^, ^14^, ^15^ However, the association of better prognosis with HPV positivity is controversial.^16^, ^17^ Currently, there is no agreement on whether HPV infection is a prognostic factor for survival associated with LSCC, as some articles have reported that HPV infection has no prognostic significance,^18^, ^19^, ^20^ whereas others considered HPV infection as a positive prognostic factors for LSCC.^21^, ^22^, ^23^, ^24^ Thus, it is essential to further understand the role of HPV in LSCC.

The test of HPV infection is based almost exclusively on molecular methods. Few HPV testing options are currently available in the clinical setting: the two most commonly used detection methods are immunohistochemical analysis (IHC) of the p16 protein and polymerase chain reaction (PCR). The sensitivity of both assays is high, but the specificity of in situ hybridization (ISH) is higher than that of both assays.^25^ Different test methods may result in inconsistent results, which may obfuscate the correlation between HPV status and prognosis in LSCC. The objective of this meta‐analysis is to better understand the association of HPV infection with prognosis in LSCC patients, specifically in terms of overall survival (OS) and disease‐free survival (DFS).

## 2 METHODS

### 2.1 Search strategy and selection criteria

This meta‐analysis was conducted according to the Preferred Reporting Items for Systematic Reviews and Meta‐Analyses (PRISMA) guidelines.^26^ For the identification of trials to be used in this meta‐analysis, we planned detailed search strategies for each database. We systematically searched for publications appearing in Pubmed Medline; Embase; Cochrane library; Web of Science; and 4 Chinese databases, that is, SinoMED, the China National Knowledge Infrastructure, WANFANG DATA, and CQVIP. We searched all of the literature up to November 12, 2018 for the combined medical subject headings (MeSH) “Papillomaviridae” and “Laryngeal Neoplasms”. The terms searched for included MeSH terms and their entry terms. The relationship between synonyms is “or”, whereas the relationship between HPV and LSCC is “and”. The search had no language restrictions. Furthermore, we selected a group with HPV infection as the exposed group, compared with the no HPV infection group.

The inclusion and exclusion criteria were set by a three‐person panel. Two researchers independently compiled the criteria for trial selection, and all disagreements were resolved by a group discussion. Titles and abstracts were screened according to the criteria. After the screening, 11 trials qualified for the meta‐analysis.^18^, ^19^, ^21^, ^22^, ^23^, ^27^, ^28^, ^29^, ^30^, ^31^, ^32^ The specific inclusion and exclusion criteria are as follows:

Trial inclusion criteria:

1. Data based on frozen tissue or formalin‐fixed tissue
2. HPV detection by PCR, ISH, or the Linear Array HPV Genotyping Test
3. The total number of cases is greater than 50
4. The number of cases in the exposed group was greater than 10
5. Population: patients with laryngeal cancer only
6. Primary outcomes: OS or DFS.

Trial exclusion criteria:

1. Case reports
2. Reviews, meta‐analyses, guidelines, and summaries
3. HPV detection by IHC
4. HPV is not the prognostic factor but risk factor
5. Without full‐text
6. Other uncorrelated studies, such as trial for the detection method, for treatment method, base researches, or animal experiments.

### 2.2 Data collection and quality assessment

A standard data extraction protocol was prepared by 2 persons. Each article was collected independently, and all relevant data were in duplicates. All disagreements were resolved through a group discussion until a consensus was reached.

Data collection from each trial included: the title, first authors, journal and year of publication, number of cases, patients inclusion criteria and age range, follow‐up period, HPV detection method, survival outcome (3‐, 5‐ and 10‐year survival rate, 3‐, 5‐ and 10‐year DFS), hazard ratio (HR) with a 95% CI, ln(HR), and SE(ln(HR)). In studies, LSCC survival was presented as Kaplan Meier curves. We used Adobe Photoshop to process the survival curve pictures, and Engauge Digitizer 4.1 software to extract the survival data, such as HR. When the difference between the extracted survival data and the value in the paper was more than 2%, data were extracted a second time.

The quality of enrolled trials was independently assessed by 2 authors using the Newcastle‐Ottawa Scale (NOS) for cohort studies. The total score ranges from 0 to 9, and scoring discrepancies were resolved by discussion in the presence of a third author as a mediator. The trials scoring at least 6 were considered high‐quality studies. The characteristics of the included studies are shown in Table 1.

**Table 1:** Characteristics of included studies

Study	Year	Country	Outcome	Sample size cases		Genotyping method	Quality score
Wang et al^32^	2016	China	3,5‐OS	25	38	PCR	6
Wang et al^30^	2014	China	3,5,10‐OS 3,5,10‐DFS	28	135	PCR	7
Duray et al^18^	2011	Belgium	3,5,10‐DFS	44	15	PCR	6
Hernandez et al^19^	2014	US	3,5‐OS	26	100	Linear Array Test	6
Barrueco et al^27^	2017	Spain	3,5,10‐OS	28	95	PCR	7
Chen et al^21^	2017	China	3,5‐OS	14	92	PCR, ISH	7
Stephen et al^29^	2012	US	3,5,10‐OS	21	56	PCR	8
Wang et al^23^	2015	China	3,5,10‐OS 3,5,10‐DFS	32	276	PCR	7
Yang et al^31^	2016	China	5‐OS 5,10‐DFS	33	163	PCR	6
Erkul et al^28^	2017	Tuekey	3,5‐DFS	19	54	PCR	7
Tong et al^22^	2018	China	3‐OS	132	79	PCR, ISH	8

Abbreviations: DFS, disease‐free survival; ISH, in situ hybridization; OS, overall survival; PCR, polymerase chain reaction.

### 2.3 Statistical analysis

We used R 3.5.3 software to analyze data from the selected studies. HR with a 95% CI was calculated to measure the survival outcome in the exposed group compared with the control group. The heterogeneity between studies was qualitatively and quantitatively examined using the chi‐squared Q test and the *I* ^2^ metric respectively. If the Q test *P‐value* was <.05 or the *I* ^2^ was <50%, suggesting heterogeneity, we calculated the HR using a random‐effects model. If no significant heterogeneity was detected, we applied a fixed‐effects model. All statistical tests were 2‐sided, and *P* < .05 suggested statistical significance. A HR >1 suggested that HPV increased the risk of poor survival, a HR <1 suggested that HPV infection was a beneficial prognostic factor, and HR = 1 suggested no significant difference in survival. Potential publication bias was assessed by a funnel plot and Egger's test, and *P*‐values higher than .05 presented there was no publication bias.

## 3 RESULTS

### 3.1 Enrolled studies

**Figure 1:**
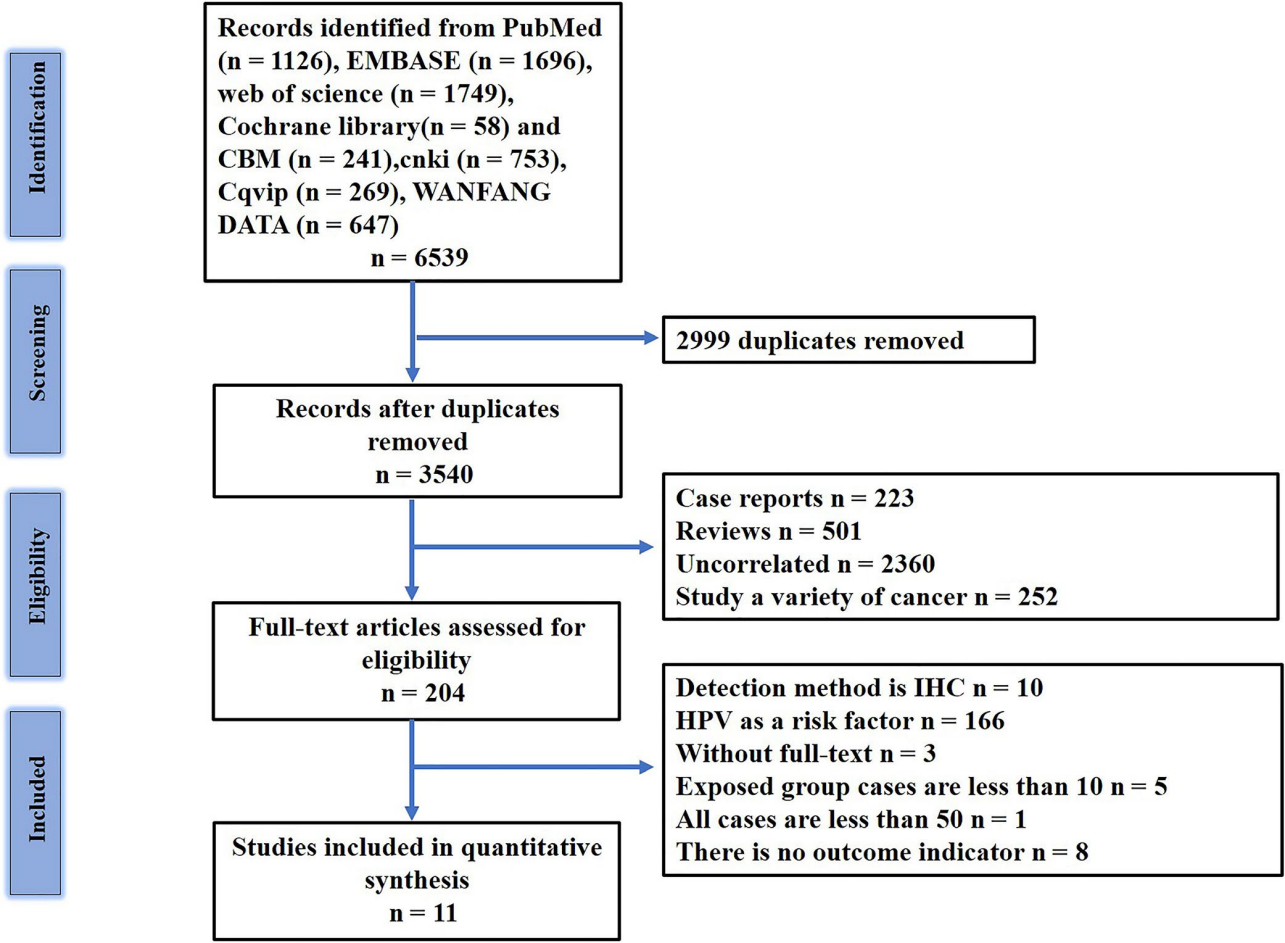
Preferred reporting items for systematic reviews and meta‐analyses flow diagram for this study

A total of 6539 potentially relevant articles were initially searched form 8 databases, of 3540 articles remained after automatically and manually deduplication. By screening the titles and abstracts, 3336 articles were excluded on the basis of the established criteria. Of the 204 articles remaining, 3 full articles could not be obtained. Furthermore, by carefully browsing 201 full‐text, 10 articles were excluded because of the detection method: in those papers, the detection method of HPV was IHC staining, whose sensitivity is lower than that of PCR. Six other articles were excluded because of the low number of cases, whereas 8 papers were excluded because of the lack of specific outcome indicators. At the end of the screening process, we selected 11 studies for the meta‐analysis (Figure 1).

The characteristics of the 11 enrolled studies are summarized in Table 1. All studies were published between 2011 and 2018. Six studies were conducted in China, 2 studies in the US, 1 study in Spain, 1 study in Belgium, and 1 study in Turkey. All of the selected studies used frozen tissue and formalin‐fixed tissue of LSCC. The detection method is PCR, ISH, or the Linear Array HPV Genotyping Test, and all studies reported at least one survival outcome. A total of 1442 LSCC cases were included in this meta‐analysis.

### 3.2 Overall survival

**Figure 2:**
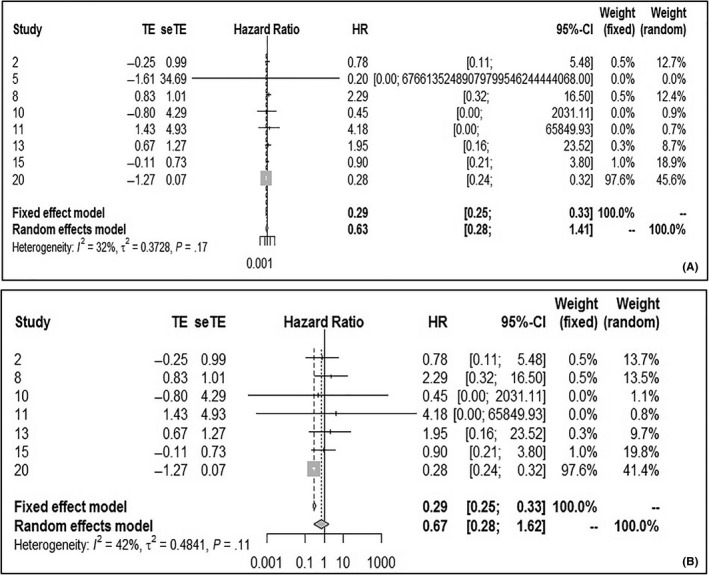
Forest plot comparing HPV‐positive to HPV‐negative LSCCs of 3‐OS. A, Including all study. B, After excluding the second study. HPV, human papilloma virus; LSCC, laryngeal squamous cell carcinoma; OS, overall survival

Eight studies examined 3‐year OS for LSCC. The pooled HR from the 8 studies was 0.29 (95% CI: 0.25‐0.33), obtained using the fixed effect model because of the low heterogeneity (Figure 2A). There was a statistically significant difference in 3‐year OS between the HPV‐negative and positive groups, with HPV infection being beneficial for survival in LSCC patients. However, in the second study,^30^ the upper limit of the 95% CI was too high. Thus, after a group discussion we decided to exclude it in order to reduce bias. After excluding the second study, the pooled HR was still 0.29 (95% CI: 0.25‐0.33; *P* heterogeneity = 0.11, *I* ^2^ = 42%) (Figure 2B). In addition, HPV infection had a statistically significant advantage in terms of 3‐year OS in LSCC patients.

**Figure 3:**
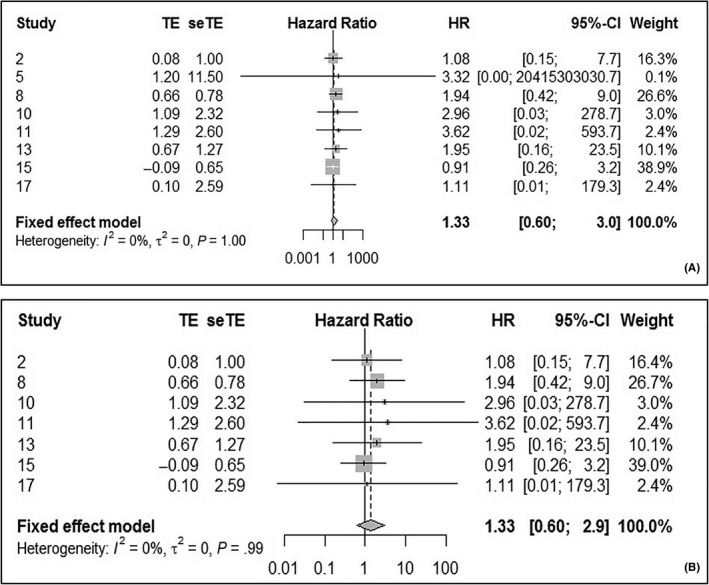
Forest plot comparing HPV‐positive to HPV‐negative LSCCs of 5‐OS. A, Including all study. B, After excluding the second study. HPV, human papilloma virus; LSCC, laryngeal squamous cell carcinoma; OS, overall survival

Eight studies examined 5‐year OS for LSCC, and no statistically significant heterogeneity (*I* ^2^ = 0%, Q‐test *P* = 1.00) was found. The pooled HR was 1.33 (95% CI: 0.60‐3.0), according to the fixed effect model (Figure 3A). There was no statistically significant difference in 5‐year OS between the HPV‐negative and ‐positive groups after the exclusion of the second study,^30^ as the pooled HR was still 1.33 (95% CI: 0.60‐3.0; *P* heterogeneity = 0.99, *I* ^2^ = 0%) (Figure 3B).

**Figure 4:**
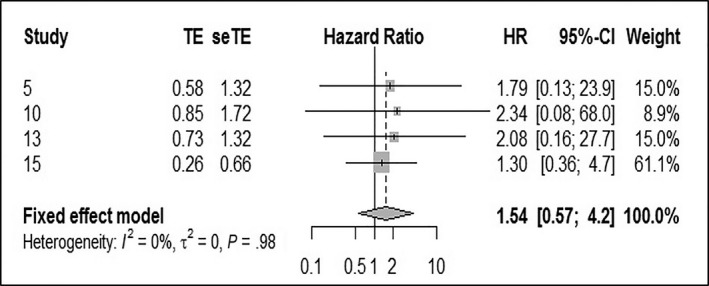
Forest plot comparing HPV‐positive to HPV‐negative LSCCs of 10‐OS. HPV, human papilloma virus; LSCC, laryngeal squamous cell carcinoma; OS, overall survival

Four studies examined 10‐year OS in relation to LSCC, and no statistically significant heterogeneity (*I* ^2^ = 0%, Q‐test *P* = .98) was found. The pooled HR was 1.54 (95% CI: 0.57‐4.2), using the fixed effect model (Figure 4). There was no statistically significant difference in 10‐year OS between the HPV‐negative and ‐positive groups.

Overall, the polled results showed that HPV infection was a favorable prognosis for 3‐year OS, while there was no statistically significant difference for 5‐ and 10‐year OS.

**Figure 5:**
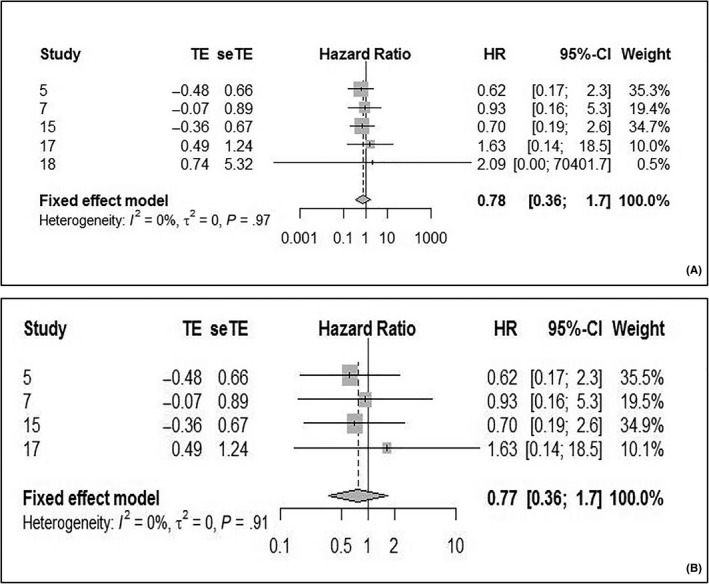
Forest plot comparing HPV‐positive to HPV‐negative LSCCs of 3‐DFS. A, Including all study. B, After excluding the last study. DFS, disease‐free survival; HPV, human papilloma virus; LSCC, laryngeal squamous cell carcinoma

The DFS is used to assess the treatment of local tumor. Five studies examined 3‐ and 5‐year DFS for LSCC, while only 3 studies examined 10‐year DFS. For the 3‐year DFS, the pooled HR was 0.78 (95% CI: 0.36‐1.7; *P* heterogeneity = 0.97, *I* ^2^ = 0%) (Figure 5A), with no statistical difference between the HPV‐positive and‐negative groups. However, in the last study,^28^ the upper limit of the 95% CI was too high. Thus, after a group discussion we decided to exclude it in order to reduce bias. The statistical results remained similar (Figure 5B).

**Figure 6:**
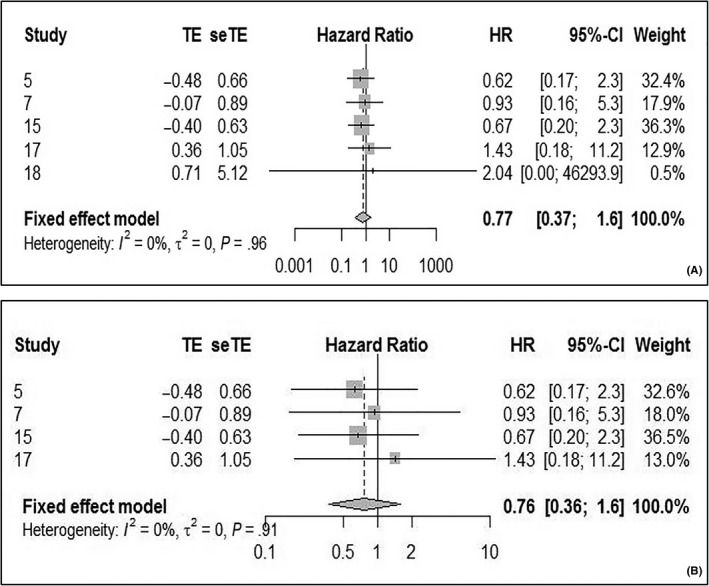
Forest plot comparing HPV‐positive to HPV‐negative LSCCs of 5‐DFS. A, Including all study. B, After excluding the last study. DFS, disease‐free survival; HPV, human papilloma virus; LSCC, laryngeal squamous cell carcinoma

**Figure 7:**
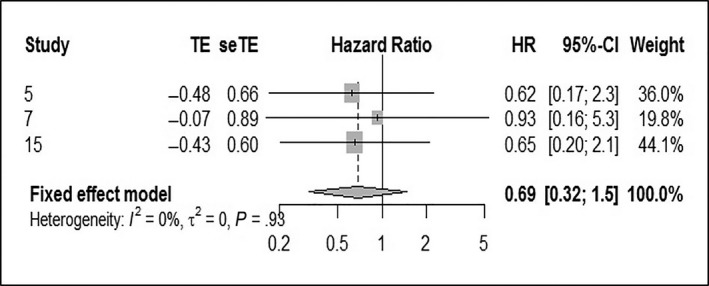
Forest plot comparing HPV‐positive to HPV‐negative LSCCs of 10‐DFS. DFS, disease‐free survival; HPV, human papilloma virus; LSCC, laryngeal squamous cell carcinoma

For the 5‐year DFS, the pooled HR was 0.77 (95% CI: 0.37‐1.6; *P* heterogeneity = 0.96, *I* ^2^ = 0%), using the fixed effect model (Figure 6A). There was no statistical significance between the HPV‐positive and ‐negative groups. Furthermore, in the last study the upper limit of 95% CI was too large, although the statistical results remained similar after its exclusion (Figure 6B). For the 10‐year DFS, the pooled HR was 0.69 (95% CI: 0.32‐1.5; *P* heterogeneity = 0.98, *I* ^2^ = 0%). There was no statistical significance between the HPV‐positive and ‐negative groups (Figure 7).

Overall, all calculated HRs were less than 1, but we observed no statistically significant difference in 5‐ and 10‐year DFS between the HPV‐negative and ‐positive groups.

Through the evaluation of publication bias by funnel plot and Egger's test, we found that the study may have publication bias. The funnel plot and Egger's test results are in the attachment.

## 4 DISCUSSION

The status of HPV infection and its impact on treatment and survival outcomes in HNC is an important research focus. As in oropharynx cancer, the infection of HPV have been reported to be a potential risk factors also for laryngeal cancer.^33^ Several studies reported that HPV infections range from 3% to 85% in LSCC.^6^, ^7^, ^9^, ^34^ The large variation of HPV prevalence could be due to changes in the geographical demographics and in the detection methods. Since HPV was discovered and reported 30 years ago, many methods for detecting HPV have been available, but each method has its own limitations. According to the central rule, the molecular detection indicators change in the different stages of HPV infection. Thus, the efficacy of HPV detection methods changes accordingly. At the DNA level, L1 and L2 fragments of HPV can be detected by ISH and PCR.^35^, ^36^ HPV 16, 18, and 33 can be genotyped using these two methods.^37^, ^38^ After the HPV viral DNA is incorporated into the host DNA, it is transcribed to produce mRNA. At the RNA level, E6 and E7 mRNAs are the main detection indicator. E6 and E7 are viral oncoproteins playing a critical role in cell transformation and malignant phenotype maintenance. The E6 protein binds to the p53 tumor suppressor gene product, whereas E7 binds to pRb, which is the phosphorylated retinoblastoma tumor suppressor protein.^39^ At the protein level, p16 is the main detection indicator and is usually detected by IHC.^40^ The sensitivity, specificity, and robustness of PCR are higher than those of IHC.^41^ Due to the low sensitivity and specificity of IHC, and some studies have indicated that p16 cannot be used as an effective indicator of HPV infection in LSCC patients.^22^, ^42^ So, the study excluded IHC as a method to detect HPV in the meta‐analysis. HPV includes more than 120 types, but the current correlation between different types and tumors is not clear, so no further analysis of HPV types was conducted in this paper. They were divided into two groups based only on whether they had HPV infection.

Currently, there are many studies investigating the relationship between different survival indicators and HPV infection. In those studies, the most frequently used survival indicators are OS and DFS. OS is measured as the time from the beginning of randomization to death from any cause. OS is considered the best endpoint to measure efficacy in clinical trials on tumors and is the preferred survival indicator. Some papers demonstrated that the OS of the HPV‐positive group was better than that of the HPV‐negative group in laryngeal cancer patients. However, other studies reported that the difference was not statistically significant.^18^ Reliable results can be obtained by performing high‐quality analysis on a high number of cases. To avoid the limitation of a small number of patients in a single original study, we combined similar studies in a meta‐analysis to increase the number of cases. In this meta‐analysis, a total of 1442 LSCC cases were included. The HR of 3‐year OS was 0.29 (95% CI: 0.25‐0.33), and the difference was statistically significant, suggesting that HPV infection is a good prognostic factor. The HR of 5‐ and 10‐year OS was 1.33 (95% CI: 0.6‐3.0) and 1.54 (95% CI: 0.57‐4.2) respectively. We observed no significant differences between HPV‐negative and ‐positive groups. From the perspective of systematic error analysis, there are several reasons explaining why the HR differences in 5‐ and 10‐year OS are not significant: (a) the number of original studies is small and the sample size included is insufficient; (b) at the later stage of following‐up, the rate of lost to follow‐up is relatively high, introducing a relatively large bias; (c) The 5‐ and 10‐year OS rate is conspicuously lower than the 3‐year OS, so the number of patients who survived was significantly reduced in 5 and 10 years, resulting in a larger bias for each individual case.

Furthermore, according to the latest NCCN guidelines, surgery or radical radiotherapy is preferred for patients with early laryngeal cancer. In this study, the 3‐year OS of the HPV‐positive group was significantly higher than the HPV‐negative group, suggesting that HPV infection is beneficial in the short‐term. This difference could be associated with the surgery techniques and methods and could be explained by the observation that HPV‐positive tumor patients have higher sensitivity to radiation therapy.^43^, ^44^, ^45^, ^46^ However, the relationship between HPV infection and radiation sensitivity needs to be further verified through improved in vitro experiments or meta‐analysis of treatment stratification with a larger sample size. In this study, the 5‐ and 10‐year OS between HPV‐positive and ‐negative groups was not statistically significant. Recurrence and metastasis of LSCC patients is most likely to occur in 3‐5 years after initial treatment. Radiotherapy and/or chemotherapy are the main treatment methods for recurrence and metastasis patients. HPV‐positive cancer cells have been reported to have an inherent resistance to chemotherapy.^47^, ^48^ So, the lack of difference in survival for LSCC patients could be explained by the use of chemotherapy and the inherent resistance of HPV‐positive tumor cells to chemotherapy agents.

DFS is measured as the length of time after treatment during which no disease is found. In some studies, it was reported that HPV infection status is an independent prognostic indicator of DFS and HPV‐positive patients have better prognosis.^23^, ^31^ However, some studies reported that, although the DFS rate of HPV‐positive patients was slightly higher than that of the HPV‐negative group, the difference was not statistically significant.^27^, ^28^ In an effort to shed light on this discrepancy, 5 original studies were combined in the meta‐analysis, including a total of 809 patients. The HR of 3‐, 5‐, and 10‐year DFS after merging was 0.77 (95% CI: 0.36‐1.7), 0.76 (95% CI: 0.36‐1.6), and 0.69 (95% CI 0.32‐1.5) respectively. All three values showed that the HPV‐positive group had a better DFS trend, but the differences were not statistically significant. However, this does not rule out the possibility that there are biological differences, suggesting that HPV infection may lead to a better local control and a lesser likelihood of relapse and metastasis, although the number of original studies and cases included in this review is too small to produce meaningful statistical results. In addition, there may be publication bias in this study, so further studies are needed to verify our results Previous studies have shown that the degree of tumor differentiation and pathological staging in oropharyngeal cancer is partly related to the infection of HPV,^49^, ^50^ with HPV‐positive tumors having a less aggressive tumor behavior. Therefore, it is necessary to conduct further multicenter studies on tumor pathological typing and local control rate in LSCC.

In recent years, an increasing amount of studies reported the relationship between HPV infection and the prognosis of patients with laryngeal cancer, but a consensus has not been reached yet. In this meta‐analysis, patients with HPV infection of LSCC have a better 3‐year OS, but we observed no significant differences in the long‐term survival rate. This result may be related to differences in the treatment plan, suggesting that future clinical research will need to evaluate different treatment outcomes. Compared with the HPV‐negative group, the HPV‐positive group had a better DFS, but there was no significant difference between the groups, suggesting that studies with a larger sample size and further studies on the pathology and local control of HPV‐positive tumors are needed.

## CONFLICTS OF INTEREST

None declared.

## AUTHORS' CONTRIBUTIONS

Conceptualization, X. J. and LH. D.; Software, B. W.; Validation, X. J. and LH. D.; Formal Analysis, B. W.; Investigation, HH. W.; Resources, HH. W.; Data Curation, Y. X.; Writing‐Original Draft Preparation, HH. W.; Writing‐Review & Editing, LB. M., Y. X., and X. J.; Funding Acquisition, X. J.. All authors read and approved the manuscript.
