# Supplementary figures and images for: Role of human papillomavirus in laryngeal squamous cell carcinoma: A meta‐analysis of cohort study
Source: Cancer Med. 2019 Nov 15;9(1):204–14. doi: 10.1002/cam4.2712 (PMC6943161; doi:10.1002/cam4.2712)

## Slide 1
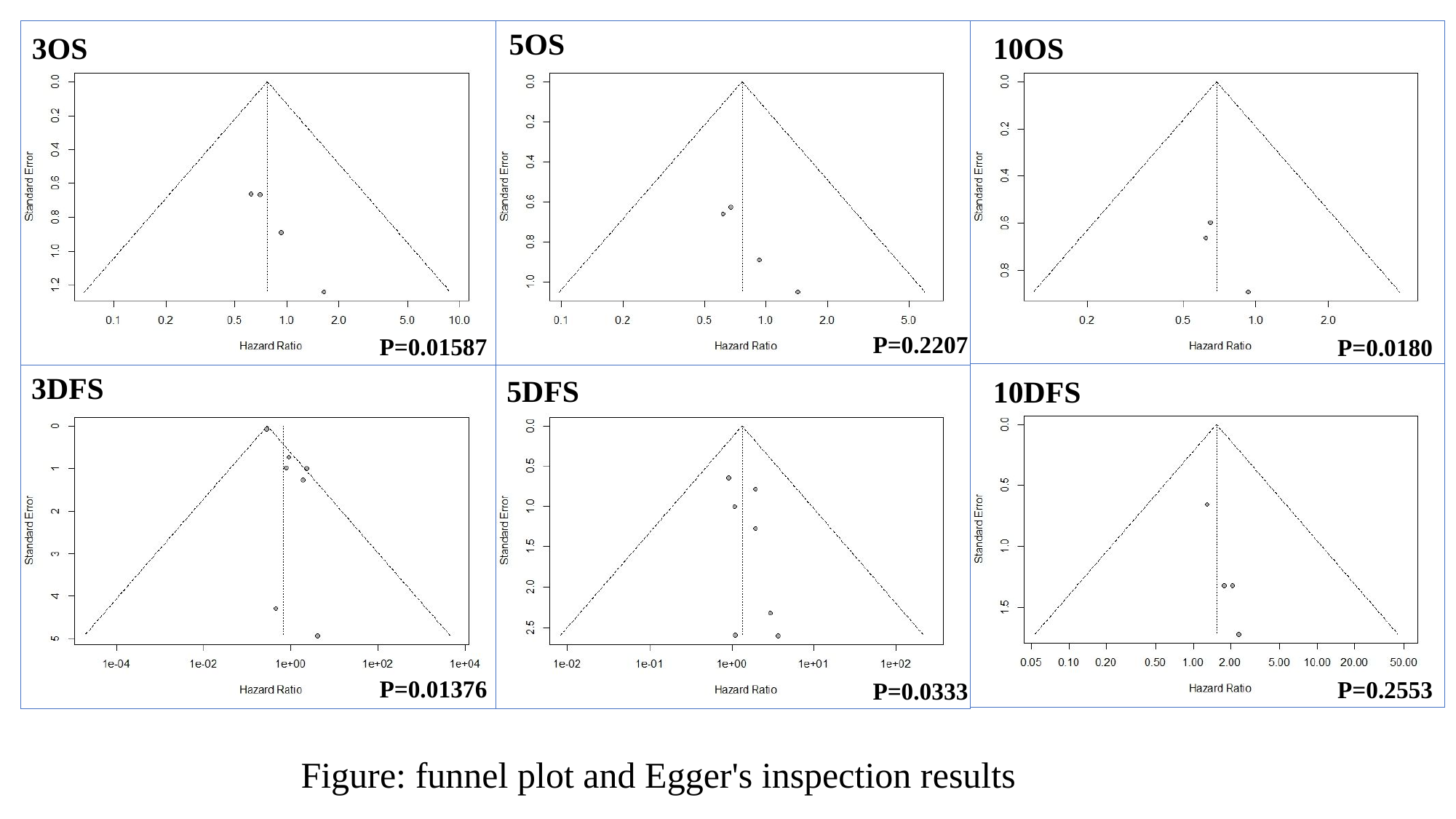

5OS
3OS
10OS
3DFS
5DFS
10DFS
P=0.2207
P=0.01587
P=0.0180
P=0.01376
P=0.2553
P=0.0333
Figure: funnel plot and Egger's inspection results

Supplement: Supplementary file 1 [file CAM4-9-204-s001.pptx]
